# Translation and evaluation of the simplified Chinese version of the rating form of IBD patient concerns

**DOI:** 10.1186/s12876-022-02503-7

**Published:** 2022-09-22

**Authors:** Jianfeng Luo, Jiamin Zhong, Haiwen Li, Shijing Zhang, Liwan Zhang, Jiang-tao Hou, Junyu Ke, Huibiao Li, Fengbin Liu, Xin-lin Chen

**Affiliations:** 1 School of Basic Medical Science, Guangzhou University of Chinese Medicine, Guangzhou, 510006 China; 2 Shenzhen Traditional Chinese Medicine Hospital, Shenzhen, 518000 Guangdong China; 3 Fuzhou Institute of Technology, Fuzhou, 350506 China; 4 The First Affiliated Hospital of Guangzhou University of Chinese Medicine, Guangzhou, 510405 China; 5 Gaozhou Hospital of Traditional Chinese Medicine Affiliated to Guangzhou University of Chinese Medicine, Gaozhou, 525200 China

**Keywords:** Inflammatory bowel disease, The RFIPC, Translation, Validation

## Abstract

**Background:**

Inflammatory bowel disease (IBD) has become a global public health problem. The prevalence of IBD in China increased annually in past two decades.

**Methods:**

This study was to translate and validate the rating form of IBD patients' concerns (RFIPC), and to describe disease-related worries and concerns of patients with IBD. The simplified Chinese version of the RFIPC was developed according to translation and back-translation procedure. Patients with IBD were consecutively enrolled from the First Affiliated Hospital of Guangzhou University of Chinese Medicine. The participants were assessed using the RFIPC and the Short Inflammatory Bowel Disease Questionnaire (SIBDQ). Internal consistency, test–retest reliability, measurement error, confirmatory factor analysis (CFA) and correlation of the RFIPC with the SIBDQ were performed to evaluate the psychometric characteristics of the RFIPC.

**Results:**

A total of 116 patients with IBD, 73 with ulcerative colitis (UC) and 43 with Crohn’s disease (CD), were enrolled in this study. Thirty-seven of them recompleted the questionnaires for the second time between 7 and 14 days after the first interview. The results of CFA indicated the original structure of the RFIPC was reasonable. Cronbach's alpha value of the RFIPC were 0.97. The intraclass correlation coefficients of four domains ranged from 0.85 to 0.92. The standard error of measurement was 7.10. The correlation coefficients between total score of the RFIPC and the SIBDQ score ranged from − 0.54 to − 0.70. Median total score of the RFIPC was 39.4 (IQR 24.0–59.3). Patients with severe symptoms reported higher scores of the RFIPC. The uncertain nature of disease, having surgery, having an ostomy bag, developing cancer, feeling out of control, being a burden on others and financial difficulties were highest concerns of patients with IBD. Comparing with patients with UC, patients with CD had more concerns of the ability to have children and being treated as different (*P* < 0.05).

**Conclusions:**

The simplified Chinese version of RFIPC is a valid and reliable tool. It could be used for assessing disease-related worries and concerns of patients with IBD in China. Specific concerns of patients with UC and CD are different, therefore, health workers should consider the specific needs of UC and CD patients.

**Supplementary Information:**

The online version contains supplementary material available at 10.1186/s12876-022-02503-7.

## Background

Inflammatory bowel disease (IBD) is a chronic and disabling disease of the gastrointestinal tract characterized by episodes of intestinal inflammation [1]. Ulcerative colitis (UC) and Crohn’s disease (CD), the two primary forms of IBD, are estimated to affect approximately 0.3% of the world’s population [2]. A modeling study predicts that there will be a 1.5-fold increase for East Asia region with 4.5 million cases, and a 1.6-fold elevation in prevalence for high‐income Asia‐Pacific and Southeast Asia regions in 2035, as compared to 2020 [3].

IBD not only damages patient's gastrointestinal tract, but also affects their mental health, causing depression and anxiety [4]. A high prevalence of psychological disorders was reported among patient with IBD in mainland China [5]. These psychological comorbidities increase disease burden and impair their quality of life directly [6–8]. Therefore, healthcare workers should be greater attention to the psychological burdens of patients with IBD.

The rating form of IBD patients' concerns (RFIPC), developed by Drossman et al. in 1991, is a commonly used instrument to evaluate IBD patients’ disease-related worries and concerns [9]. It has been translated into 10 languages since its publication [10–17]. Currently, the RFIPC has been widely used in cross-sectional and prospective longitudinal studies [18, 19]. However, the RFIPC has not been translated into Chinese. The study aimed to translate the RFIPC into simplified Chinese and to evaluated its psychometric properties. Furthermore, we attempted to investigate disease-related worries and concerns among patients with IBD in mainland China.

## Patients and methods

### Patients

From June 2020 to June 2021, Chinese-speaking patients with IBD were consecutively invited from the First Affiliated Hospital of Guangzhou University of Chinese Medicine. Patients were eligible if they were between 16 and 75 years old, with an established diagnosis of UC or CD both by endoscopy and histological examination, classified according to the Montreal classification of inflammatory bowel disease [20]. The exclusion criteria were (1) patients with IBD who refused to participate in the study; (2) patients with severe cognitive impairment who could not understand the questionnaire; (3) patents with co-existent diseases (such as chronic heart failure, chronic renal failure, malignant tumours etc.) or neuropsychiatric disorders that can affect the results of the study. Trained researchers had face-to-face interview with eligible patients and invited them to participate in the study. Participants were asked to fill a set of questionnaires on the spot. The participants were asked to fill in the RFIPC and questionnaire about major symptoms (QMS) of IBD once again if they returned for further consultations between 7 and 14 days. All questionnaires were self-administered. Researchers would help explained the questions to participants when necessary.

### Questionnaires

#### Demographic characteristics and medical information

Demographic characteristics of participants included gender, age, marital status, level of education, smoking and drinking. Medical information was about disease type, disease location, and the QMS of IBD. The QMS was regarded as an assessment of disease activity, including severity of abdominal pain, frequency of stool, level of fatigue, degree of weight loss. The questionnaire was self-administrated and recommended as an efficacy evaluation for treating colitis in *Development of clinical trial of new drugs of traditional Chinese medicines* published by the National Medical Product Administration of China [21]. Each symptom was rated on a four-point Likert scale from 0 (symptom not present) to 3 (severe). A higher score indicated a more severe symptom.

#### The simplified Chinese version of the RFIPC

The RFIPC is a 25-item questionnaire with each item scoring on a horizontal visual analogue scale 0–100 mm (0 = Not at all, 100 = A great deal). In the original questionnaire, 22 of 25 items were divided into four factors: disease impact, complications, sexual intimacy, and body stigma. An overall mean score of all items was as “sum score” [9].

After obtaining license from the original authors, the translation and back-translation process of the RFIPC were conducted in line with Brislin’s guidelines [22, 23]. First, two bilingual (Chinese and English) native experienced researchers translated the questionnaire from English to simplified Chinese independently. Then, the translation coordinator compared the two simplified Chinese version of the RFIPC and conduct a reconciliation process to produce the first draft. Second, the first draft of RFIPC was back-translated into English by two other bilingual researchers who were not involved in translation process. Thereafter, the coordinator discussed any discrepancies between the original source and the back-translated questionnaire with both forward and back translators. The subject “您的” (meaning “your”) was added to item “attractiveness”, “energy level”, “ability to perform sexually” for ease of understanding. Finally, the final version of the RFIPC was formed.

Cross-cultural adaptation of the final version of RFIPC was conducted using a pre-test. The pre-test involving 6 patients with IBD and 6 healthy controls aimed to identify any ambiguity in the items and wordings of the questionnaire. All subjects participated in the pre-testing completed the questionnaire in less than 10 min. They reported no difficulties in reading, understanding or answering the RFIPC. No change was made to the questionnaire after the pre-testing.

#### The short inflammatory bowel disease questionnaire (SIBDQ)

The SIBDQ is a short version of the Inflammatory Bowel Disease Questionnaire, and is used as a health-related quality-of-life measure of patients with IBD. The SIBDQ includes 10 questions grouped into 4 domains (bowel symptoms, systemic symptoms, social function, emotional function). All items are rated on a 7-point Likert scale (1 = all the time, 7 = never). The total score ranges from 10 to 70. A higher score indicates a better quality of life [24]. A simplified Chinese version of SIBDQ has been proofed to be a quick and reliable quality-of-life instrument for patients with IBD in mainland China [25].

### Statistical analysis

All data from the questionnaires were pooled into Microsoft Office Excel 2016. The quality of a questionnaire’s measurement properties was evaluated by the Consensus-based Standards for the selection of health Measurement Instruments (COSMIN) [26]. For normally distributed continuous variables, means and standard deviations (SD) were presented. Median and interquartile range (IRQ) values were used to describe the nonnormally distributed continuous variables. Mann–Whitney U test or Kruskal–Wallis H test were used to compare medians of nonnormally distributed variables. The frequencies of categorical variables were compared using Pearson’s Chi-square test or Fisher's exact test. All statistical tests were considered significant with *P* ≤ 0.05. We performed all analyses using the Statistical Package for Social Sciences (SPSS 25.0, SPSS Inc., Chicago, IL, USA) and the IBM AMOS 24.0.

Confirmatory factor analysis (CFA) and Spearman’s correlation coefficient (*r*_*s*_) between the RFIPC and the SIBDQ was performed to evaluate validity. The goodness of fit of CFA model was assessed using root mean square error of approximation (RMSEA) and comparative fit index (CFI). RMSEA > 0.06, and CFI > 0.9 were recommended.

Reliability was tested by internal consistency and test–retest reliability. Cronbach's alpha coefficient values > 0.7 indicated strong internal consistency. The test–retest reliability was assessed using intraclass correlation coefficients (ICC). An ICC > 0.7 was considered as good reliability.

Measurement error was calculated using standard error of measurement (SEM) [27].

Scores of each domain for patients with different disease types and severity of major symptoms were compared in order to understand how disease types and disease activity affect patients’ worries and burdens. The score of each item was compared in order to identify differences of concerns between patients with UC and CD.

## Results

### Participant characteristics

A total of 119 eligible patients were enrolled. Two of them refused to take part in the study. One patient was excluded because of missing data of medical information. At last, 116 patients were included for analysis. Among them, 37 patients were included for a second measurement. The mean age of 116 patients were 37.8 years (SD = 13.5 years), and 35.3% of them were women. The majority of patients had college degree and above (58.6%). More details of demographic characteristics were presented in Table 1.

**Table 1:** The characteristics of the included patients

	IBD* (n = 116)	Test–retest (n = 37)	*P*-value
Sex			0.213^a^
Males (%)	75 (64.7)	28 (75.7)	
Females (%)	41 (35.3)	9 (24.3)	
Age(mean ± SD)	37.8 ± 13.5	36.3 ± 13.2	0.550^b^
Level of education			0.841^a^
High school diploma or less (%)	48 (41.4)	16 (43.2)	
College degree and above (%)	68 (58.6)	21 (56.8)	
Marital status			0.368^a^
Married (%)	75 (64.7)	21 (56.8)	
Single (%)	41 (35.3)	16 (43.2)	
Smoking			0.354^c^
Non-smoker (%)	73 (62.9)	23 (62.2)	
Ex-smoker (%)	33 (28.4)	13 (35.1)	
Smoker (%)	10 (8.6)	1 (2.7)	
Drinking			0.676^a^
Yes (%)	29 (25.0)	8 (21.6)	
No (%)	87 (75.0)	29 (78.4)	
Diagnosis			0.502 ^a^
UC (%)	73 (62.9)	21 (56.8)	
CD (%)	43 (37.1)	16 (43.2)	
Disease location of UC			0.483^c^
Proctitis (%)	38 (33.0)	14 (37.8)	
Left-sided colitis (%)	21 (18.3)	4 (10.8)	
Pancolitis (%)	14 (12.2)	3 (8.1)	
Disease location of CD			0.492^c^
Small bowel (%)	25 (22.3)	10 (29.4)	
Colon (%)	7 (6.2)	4 (11.8)	
Colon + small bowel (%)	11 (9.8)	2 (5.9)	
Diarrhea, times/day			0.630^d^
Never	49 (42.2)	17 (45.9)	
< 3	40 (34.5)	13 (35.1)	
3–6	22 (19.0)	5 (13.5)	
> 6	5 (4.3)	2 (5.4)	
Bloody stools			0.755^d^
Never	73 (62.9)	22 (59.5)	
Few	35 (30.2)	13 (35.1)	
Mostly	5 (4.3)	0 (0)	
Entire	3 (2.6)	2 (5.4)	
Abdominal pain			0.998^d^
No	19 (16.4)	7 (18.9)	
Mild	57 (49.1)	16 (43.2)	
Moderate	25 (21.6)	10 (27.0)	
Severe	15(12.9)	4 (10.8)	
Weight loss			0.959^d^
No	39 (33.6)	12 (32.4)	
Mild	25 (21.6)	9 (24.3)	
Moderate	22 (19.0)	6 (16.2)	
Severe	30 (25.8)	10 (27.0)	

*IBD* inflammatory bowel disease, *UC* ulcerative colitis, *CD* Crohn’s disease

^a^*P*-value: Pearson’s Chi-Square; ^b^*P*-value: *t*-test; ^c^*P*-value: Likelihood ratio; ^d^*P*-value: Kruskal Wallis H test

### Validity

**Fig. 1:**
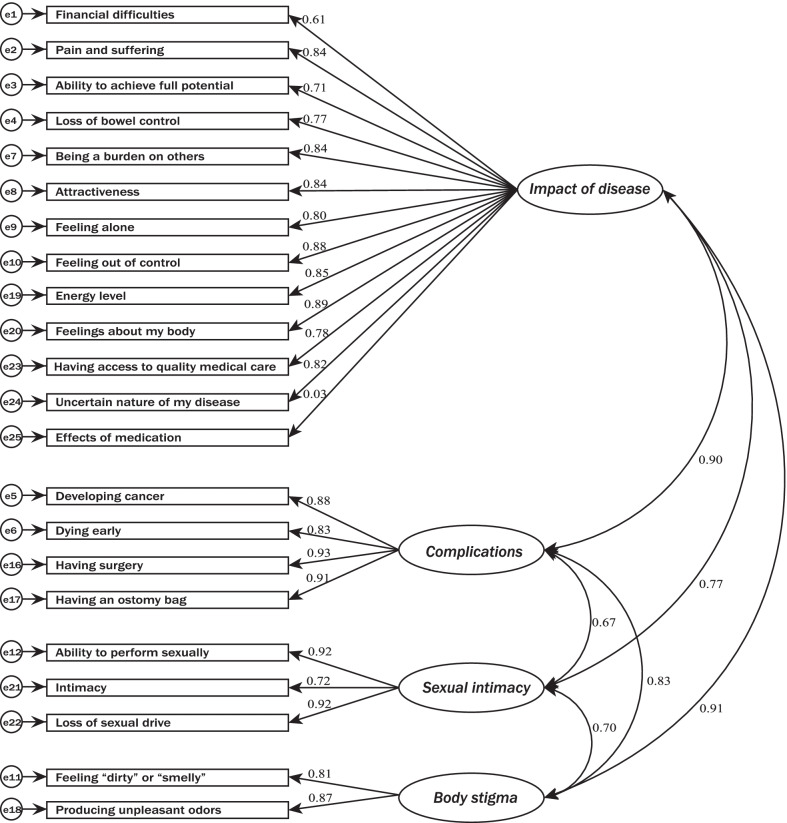
The CFA model of the RFIPC (standardised factor loadings)

The CFA of the original model (including 4 factors, Fig. 1) was performed. Chi-square, RMSEA, and CFI of model fit were 774.01, 0.16 and 0.80, respectively. These results suggested that original structure of the RFIPC was reasonable.

Total score of the RFIPC was moderate to high negatively correlated with the total SIBDQ score (*r*_*s*_ = − 0.67,* P* < 0.001). The results of correlation between domains of RFIPC and SIBDQ were shown in Table 2. “Impact of disease” of the RFIPC had strong negative correlation with “emotional function” of the SIBDQ (*r*_*s*_ = − 0.88).

**Table 2:** Spearman’s correlation coefficients (*r*_*s*_) between the RFIPC and the SIBDQ

The RFIPC	Cronbach’s alpha	Correlation with the SIBDQ				
		Bowel symptoms	Social function	Emotional function	Systemic symptoms	Total SIBDQ
Impact of disease	0.96	− 0.66	− 0.62	− 0.88	− 0.57	− 0.70
Complications	0.94	− 0.51	− 0.53	− 0.39	− 0.44	− 0.54
Sexual intimacy	0.89	− 0.56	− 0.49	− 0.47	− 0.48	− 0.57
Body stigma	0.83	− 0.56	− 0.51	− 0.40	− 0.45	− 0.56
Total RFIPC	0.97	− 0.64	− 0.61	− 0.54	− 0.55	− 0.67

### Reliability

The Cronbach’s alpha value for the RFIPC indicated an acceptable level of internal consistency (α = 0.97). Cronbach’s alpha for each domain of the RFIPC ranged from 0.83 to 0.96 (Table 2), indicating good internal reliability.

Thirty-seven participants, who returned to the hospital during 7–14 days after the first interview, filled in the RFIPC and the QMS for the second time. ICC of domains ranged from 0.85 to 0.92. The RFIPC showed high test–retest reliability (Table 3). They reported minor changes (*P* > 0.05) of major symptoms during those days (Additional file 1: Table S1).

**Table 3:** Test–retest reliability for 37 IBD patients (16 with CD and 21 with UC)

Domain	Visit 1	Visit 2	*P-value	ICC	95%CI
	Median [IQR]	Median [IQR]			
Impact of disease	42.5 [22.2–57.2]	43.1 [22.5–63.1]	0.213	0.88	0.82–0.95
Complications	60.0 [28.8–71.3]	45.0 [25.0–73.8]	0.516	0.92	0.85–0.96
Sexual intimacy	26.7 [15.0–48.3]	23.3 [10.0–53.3]	0.847	0.85	0.72–0.92
Body stigma	30.0 [15.00–57.5]	35.0 [10.0–70.0]	0.150	0.86	0.75–0.93

*UC* ulcerative colitis, *CD* Crohn’s disease, *ICC* intraclass correlation coefficient, *CI* confidence interval

*P-value: Wilcoxon Signed Ranks test

### Measurement error

The standard deviation of the sample (both of the test and retest administration were pooled together) was 23.68. ICC of the total scores of the RIFPC was 0.91. The SEM of the RFIPC was 7.10.

### Worries and concerns

Median total score of the RFIPC was 39.4 (IQR 24.0–59.3). Item “uncertain nature of disease” was the primary concern, follow by “having surgery”, “having an ostomy bag”, “developing cancer”, “feeling out of control”, “being a burden on others” and “financial difficulties”. Patients with severe symptoms, such as bloody stool and abdominal pain, reported higher scores in all 4 domains of RFIPC (Table 4). No significant differences of total score and domain scores were found between patients with UC and CD. However, patients with CD had higher concerns of financial difficulties, the ability to have children, and being treated as different, when comparing to patients with UC (*P* < 0.05) (Table 5).

**Table 4:** The scores of RFIPC domains for patients with different disease types and severity of major symptoms

	Impact of disease	Complications	Sexual intimacy	Body stigma
	Median [IQR]	Median [IQR]	Median [IQR]	Median [IQR]
CD (n = 43)	45.0 [22.5–69.4]	50.0 [22.5–90.0]	30.0 [10.0–50.0]	30.0 [15.0–60.0]
UC (n = 73)	38.1 [24.4–55.0]	50.0 [28.8–73.8]	23.6 [13.3–45.0]	30.0 [15.0–47.5]
^a^*P*-value	0.289	0.714	0.383	0.556
Diarrhea, times/day				
Never (n = 49)	29.4 [19.4–50.6]	32.5 [20.0–65.0]	20.0 [10.0–33.3]	20.0 [10.0–35.0]
< 3 (n = 44)	41.6 [26.6–66.3]	61.3 [32.5–90.0]	30.0 [16.7–55.0]	32.5 [15.0–55.0]
3–6 (n = 22)	49.4 [37.5–58.1]	55.0 [35.0–77.5]	36.7 [16.7–46.7]	37.5 [25.0–50.0]
> 6 (n = 5)	70.6 [64.4–83.1]	77.5 [75.0–100.0]	40.0 [40.0–100.0]	100.0 [60.0–100.0]
^b^*P*-value	0.003	0.003	0.050	0.001
Bloody stool				
No (n = 73)	28.8 [20.0–54.4]	37.5 [20.0–72.5]	20.0 [10.0–40.0]	25.0 [10.0–40.0]
Mild (n = 35)	50.0 [40.6–57.5]	60.0 [45.0–86.3]	40.0 [20.0–55.0]	40.0 [20.0–55.0]
Moderate (n = 5)	83.1 [58.1–83.1]	100.0 [55.0–100.0]	40.0 [35.0–10.00]	40.0 [35.0–100.0]
Severe (n = 3)	64.4 [48.4–77.2]	77.5 [76.3–88.8]	55.0 [55.0–77.5]	55.0 [55.0–77.5]
^b^*P*-value	0.001	0.002	0.006	0.003
Abdominal pain				
No (n = 19)	21.9 [17.2–37.2]	30.0 [13.8–43.8]	10.0 [10.0–31.7]	15.0 [10.0–20.0]
Mild (n = 57)	35.6 [21.9–50.6]	45.0 [22.5–75.0]	20.0 [10.0–30.0]	25.0 [15.0–40.0]
Moderate (n = 25)	50.0 [37.5–68.1]	55.0 [42.5–72.5]	43.3 [20.0–56.7]	40.0 [30.0–55.0]
Severe (n = 15)	66.9 [58.4–85.6]	82.5 [76.3–100.0]	63.3 [38.3–78.3]	55.0 [47.5–90.0]
^b^*P*-value	< 0.001	< 0.001	< 0.001	< 0.001
Weight loss				
No (n = 39)	29.4 [19.1–43.4]	37.5 [21.3–68.8]	13.3 [10.0–30.0]	20.0 [10.0–30.0]
Mild (n = 25)	31.3 [22.5–45.6]	30.0 [20.0–52.5]	16.7 [16.7–26.7]	25.0 [15.0–40.0]
Moderate (n = 22)	49.4 [35.0–69.4]	55.0 [45.0–90.0]	43.3 [20.0–53.3]	37.5 [15.0–55.0]
Severe (n = 30)	55.0 [43.8–70.6]	75.0 [50.0–100.0]	40.0 [26.7–70.0]	52.5 [30.0–75.0]
^b^*P*-value	< 0.001	< 0.001	< 0.001	< 0.001

*UC* ulcerative colitis, *CD* Crohn’s disease

^a^*P*-value: Mann–Whitney U test; ^b^*P*-value: Kruskal Wallis H test

**Table 5:** Comparison of worries and concerns between patients with UC and CD

Item	Total (n = 113)	UC (n = 73)	CD (n = 43)	*P-value
	Median [IQR]	Median [IQR]	Median [IQR]	
Financial difficulties	50.0 [20.0–70.0]	30.0 [20.0–60.0]	50.0 [30.0–80.0]	0.016
Pain and suffering	30.0 [20.0–60.0]	30.0 [20.0–60.0]	40.0 [20.0–70.0]	0.256
Ability to achieve full potential	40.0 [20.0–60.0]	40.0 [20.0–60.0]	40.0 [20.0–70.0]	0.467
Loss of bowel control	30.0 [20.0–60.0]	30.0 [20.0–50.0]	40.0 [20.0–70.0]	0.277
Developing cancer	50.0 [20.0–90.0]	50.0 [30.0–90.0]	50.0 [20.0–85.0]	0.668
Dying early	40.0 [20.0–70.0]	40.0 [20.0–60.0]	40.0 [20.0–80.0]	0.729
Being a burden on others	50.0 [30.0–80.0]	40.0 [30.0–70.0]	60.0 [30.0–85.0]	0.148
Attractiveness	30.0 [20.0–60.0]	30.0 [20.0–60.0]	50.0 [20.0–70.0]	0.263
Feeling alone	30.0 [20.0–50.0]	30.0 [20.0–50.0]	20.0 [20.0–55.0]	0.716
Feeling out of control	50.0 [30.0–72.5]	50.0 [30.0–70.0]	50.0 [25.0–80.0]	0.641
Feeling “dirty” or “smelly”	30.0 [10.0–50.0]	30.0 [20.0–50.0]	30.0 [10.0–60.0]	0.940
Ability to perform sexually	30.0 [10.0–50.0]	30.0 [10.0–50.0]	30.0 [10.0–65.0]	0.503
Ability to have children	20.0 [10.0–50.0]	10.0 [10.0–40.0]	30.0 [10.0–75.0]	0.012
Passing the disease to others	20.0 [10.0–50.0]	20.0 [10.0–50.0]	20.0 [10.0–60.0]	0.641
Being treated as different	30.0 [10.0–40.0]	20.0 [10.0–40.0]	30.0 [20.0–50.0]	0.016
Having surgery	50.0 [27.5–82.5]	50.0 [30.0–80.0]	50.0 [25.0–95.0]	0.307
Having an ostomy bag	50.0 [20.0–90.0]	50.0 [20.0–90.0]	70.0 [25.0–100.0]	0.266
Producing unpleasant odors	20.0 [10.0–52.5]	20.0 [10.0–50.0]	30.0 [10.0–60.0]	0.203
Energy level	40.0 [20.0–70.0]	40.0 [20.0–60.0]	40.0 [20.0–75.0]	0.712
Feelings about my body	40.0 [20.0–70.0]	40.0 [20.0–60.0]	30.0 [20.0–75.0]	0.954
Intimacy	20.0 [10.0–50.0]	20.0 [10.0–40.0]	20.0 [10.0–60.0]	0.355
Loss of sexual drive	20.0 [10.0–50.0]	20.0 [10.0–40.0]	20.0 [10.0–55.0]	0.343
Having access to quality medical care	40.0 [20.0–60.0]	40.0 [20.0–60.0]	30.0 [20.0–75.0]	0.682
Uncertain nature of my disease	60.0 [30.0–90.0]	60.0 [30.0–90.0]	70.0 [30.0–90.0]	0.961
Effects of medication	50.0 [20.0–80.0]	50.0 [20.0–80.0]	50.0 [30.0–80.0]	0.452
Total score	39.4 [24.0–59.3]	38.4 [24.8–54.4]	42.0 [21.4–68.4]	0.360

*UC* ulcerative colitis, *CD* Crohn’s disease, *IQR* interquartile range

*P-value: Mann–Whitney U test

## Discussion

The incidence and prevalence of IBD in China is increasing annually as a result of a rapid society transition culminating in a westernized environment [28]. The RIFPC is a quality-of-life instrument specified in measuring disease-related worries and concerns of IBD patients. To date, at least 10 different translated version of the RFIPC have been applied in research worldwide [10–17]. The RIFPC would help assessing disease-related worries and concerns of IBD patients in China.

The simplified Chinese version of the RFIPC is valid and reliable. The procedure of translation followed the guidelines of Brislin’s translation model [22, 23] and the validation study was carried out under guidance of the COSMIN [26]. Our results of CFA showed that the original structure of the RFIPC was appropriate. However, the model fit of this model was at the lower acceptable limit. Similarly, the authors of the Swedish version reported that the 4-factor model was a substantive improvement over the single-factor model, but still remain inadequate [29]. Both internal consistency and test–retest reliability of the RFIPC were good (Cronbach’s alpha = 0.97, ICC of domains: 0.85–0.92). These results were in accordance with some previous validation studies. For example, Cronbach’s alpha of the Greek version was 0.95, and ICC were 0.77–0.93 [15]. Cronbach’s alpha and ICC of the Swedish version were 0.95 and 0.79 respectively [12].

The importance of specific concerns varies among countries for the difference of social, cultural, and/or economic [13]. In this study, the uncertain nature of disease, having surgery, having an ostomy bag, developing cancer, feeling out of control, being a burden on others and financial difficulties were the highest concerns of patients with IBD were. In Greek, the unknown nature of disease was the primary concern, followed by feeling out of control, having access to quality medical care, fear of side effects (of medication), and energy level [15]. For patients with IBD in Spain, the five highest rated concerns were effects of medication, having an ostomy bag, the uncertain nature of disease, energy level, and developing cancer [16].

Even though UC and CD have similar burden and goals for treatment [30], there are some differences of disease-related worries and concerns. For patients with CD, they reported higher concerns of “ability to have children” and “being treated as different” when comparing with patients with UC (*P* < 0.05). Besides, “having an ostomy bag” was rated the highest score, since patients with CD may be at higher risk of needing a permanent ostomy, which was associated with reduced social role satisfaction [31]. Concern of “financial difficulties” seemed to be higher for CD patients, however, we did not investigate family economic conditions of the participants and their economic burden of IBD.

There were some limitations of our study. (1) Only 116 patients participated in this study. The results may be insufficient to generalize all the patients with IBD in mainland China. (2) The QMS questionnaire, which was solely used in China, was used as a measure of clinical activity in this study. Our results would be more convincing if the partial Mayo score and the Harvey-Bradshaw index, etc. were applied for assessing disease activity. (3) Content validity analysis was not included in this study. But, by using the RFIPC, the physician may be able to identify and rank concerns that may not otherwise be asked, or which patients may not volunteer, but which are still important [9]. This finding could be a support to the content validity of the RFIPC. (4) Criterion validity analysis was not performed because no gold standards existed for quality-of-life instruments [32]. (5) The time period between the initial and the repeated administration was set between 7 to 14 days to ensure clinical condition of the participants did not change during those days. Though Terwee et al. point out that the 1 or 2 weeks will be appropriate [33], recall bias should be taken into account. A further study involving a larger sample size, as well as disease activity index, family economic conditions and costs, is needed in order to provide a more precise result.

## Conclusions

The simplified Chinese version of RFIPC was translated according to the standard process for translating instruments. The RFIPC is a valid and reliable tool. It could be used for assessing disease-related worries and concerns of patients with IBD in China. The RFIPC was recommended by patients with IBD. Specific concerns of patients with UC and CD are different, therefore, health workers should consider the specific needs of UC and CD patients when working out strategies for treatment and disease management.

## Supplementary Information

**Additional file 1.** **Table S1.** Comparison of the major symptoms of the 37 test-retested IBD patients (16 with CD and 21 with UC).

## Data Availability

The authors declare that all data of this study are available within the main text and supplementary file, or from the corresponding author on reasonable request.

## Abbreviations

IBD: Inflammatory bowel disease
UC: Ulcerative colitis
CD: Crohn’s disease
RFIPC: Rating form of IBD patients' concerns
SIBDQ: The Short Inflammatory Bowel Disease Questionnaire
QMS: Questionnaire about major symptoms
CFA: Confirmatory factor analysis
CI: Confidence interval
COSMIN: Consensus-based Standards for the selection of health Measurement Instruments
SD: Standard deviation
IRQ: Interquartile range
RMSEA: Root Mean Square Error of Approximation
CFI: Comparative Fit Index
ICC: Intraclass correlation coefficient
SEM: Standard error of measurement
