# Supplementary material for: Translation and evaluation of the simplified Chinese version of the rating form of IBD patient concerns
Source: BMC Gastroenterol. 2022 Sep 22;22:426. doi: 10.1186/s12876-022-02503-7 (PMC9494765; doi:10.1186/s12876-022-02503-7)
Supplement: Supplementary file 1 — Additional file 1. Table S1. Comparison of the major symptoms of the 37 test-retested IBD patients (16 with CD and 21 with UC). [file 12876_2022_2503_MOESM1_ESM.docx]

**Table S1 Comparison of the major symptoms of the 37 test-retested IBD patients (16 with CD and 21 with UC)**

| Symptoms | Visit 1  n (%) | Visit 2  n (%) | **P*-value |
| --- | --- | --- | --- |
| Diarrhea, times/day |  |  | 0.593 |
| Never | 17 (45.9) | 17 (45.9) |  |
| < 3 | 13 (35.1) | 13 (35.1) |  |
| 3 – 6 | 5 (13.5) | 7 (18.9) |  |
| > 6 | 2 (5.4) | 0 (0.0) |  |
| Bloody stool |  |  | 0.414 |
| No | 22 (59.5) | 19 (51.4) |  |
| Mild | 13 (35.1) | 15 (40.5) |  |
| Moderate | 0 (0.0) | 3 (8.1) |  |
| Severe | 2 (5.4) | 0 (0.0) |  |
| Abdominal pain |  |  | 0.124 |
| No | 7 (18.9) | 12 (32.4) |  |
| Mild | 16 (43.2) | 15 (40.5) |  |
| Moderate | 10 (27.0) | 6 (16.2) |  |
| Severe | 4 (10.8) | 4(10.8) |  |
| Weight loss |  |  | 0.582 |
| No | 12 (32.4) | 16 (43.2) |  |
| Mild | 9 (24.3) | 6 (16.2) |  |
| Moderate | 6 (16.2) | 8 (21.6) |  |
| Severe | 10(27.0) | 7(18.9) |  |

IBD: inflammatory bowel disease; UC: ulcerative colitis; CD: Crohn’s disease.

**P*-value: Wilcoxon Signed Ranks Test
